# Dual Window Pattern Recognition Classifier for Improved Partial-Hand Prosthesis Control

**DOI:** 10.3389/fnins.2016.00058

**Published:** 2016-02-23

**Authors:** Eric J. Earley, Levi J. Hargrove, Todd A. Kuiken

**Affiliations:** ^1^Center for Bionic Medicine, Rehabilitation Institute of ChicagoChicago, IL, USA; ^2^Department of Biomedical Engineering, Northwestern UniversityEvanston, IL, USA; ^3^Department of Physical Medicine and Rehabilitation, Northwestern University Feinberg School of MedicineChicago, IL, USA

**Keywords:** pattern recognition, electromyography (EMG), partial-hand prosthesis, myoelectric control, intrinsic hand muscles

## Abstract

Although partial-hand amputees largely retain the ability to use their wrist, it is difficult to preserve wrist motion while using a myoelectric partial-hand prosthesis without severely impacting control performance. Electromyogram (EMG) pattern recognition is a well-studied control method; however, EMG from wrist motion can obscure myoelectric finger control signals. Thus, to accommodate wrist motion and to provide high classification accuracy and minimize system latency, we developed a training protocol and a classifier that switches between long and short EMG analysis window lengths. Seventeen non-amputee and two partial-hand amputee subjects participated in a study to determine the effects of including EMG from different arm and hand locations during static and/or dynamic wrist motion in the classifier training data. We evaluated several real-time classification techniques to determine which control scheme yielded the highest performance in virtual real-time tasks using a three-way ANOVA. We found significant interaction between analysis window length and the number of grasps available. Including static and dynamic wrist motion and intrinsic hand muscle EMG with extrinsic muscle EMG significantly reduced pattern recognition classification error by 35%. Classification delay or majority voting techniques significantly improved real-time task completion rates (17%), selection (23%), and completion (11%) times, and selection attempts (15%) for non-amputee subjects, and the dual window classifier significantly reduced the time (8%) and average number of attempts required to complete grasp selections (14%) made in various wrist positions. Amputee subjects demonstrated improved task timeout rates, and made fewer grasp selection attempts, with classification delay or majority voting techniques. Thus, the proposed techniques show promise for improving control of partial-hand prostheses and more effectively restoring function to individuals using these devices.

## Introduction

As of 2005, there were an estimated 455,000 individuals in the United States living with partial-hand amputations (Ziegler-Graham et al., 2008), with over 14,500 new cases occurring each year (Dillingham et al., 2002). Less than half of all partial-hand amputees are able to return to their previous employment, and those who do must generally make major changes to their work-related tasks (Burger et al., 2007). Cosmetic prostheses, though commonly used, provide only limited functionality. Powered myoelectric prostheses, controlled with surface electromyographic (EMG) signals have been used clinically by higher-level upper-limb amputees since the 1970s (Feeny and Hagaeus, 1970; Yamada et al., 1983; Parker and Scott, 1986), but have only recently become available to partial-hand amputees (Weir and Grahn, 2000; Gow, 2014). These prostheses can provide functional hand-grasps not available in earlier myoelectric prostheses, though they are still restricted by inadequate control methods (Phillips et al., 2012).

Touch-sensitive resistors and conventional myoelectric control strategies that rely on EMG signal amplitude have been used to control partial-hand prosthesis movements. EMG signals are acquired from either extrinsic hand muscles (located in the forearm) or intrinsic hand muscles (located in the hand). Control methods utilizing extrinsic hand muscle EMG often restrict or immobilize the wrist, which leads to a loss of functionality for the user (Bertels et al., 2009; Lang, 2011). However, due to the importance of the wrist in the completion of daily activities, the preservation of wrist motion is a primary design goal of partial-hand prostheses (Lake, 2009; Uellendahl and Uellendahl, 2012). Though conventional control using intrinsic hand muscle EMG is not compromised by wrist motion, users must operate an unintuitive switching mechanism to control more than one prosthetic function. Furthermore, EMG from intrinsic muscles can be very challenging to comfortably and robustly capture using electrodes mounted within the patient's socket.

Compared to conventional amplitude-based control, pattern recognition of forearm muscle EMG provides intuitive control of more prosthetic functions using physiologically appropriate contractions (Hudgins et al., 1993; Englehart and Hudgins, 2003; Kuiken et al., 2009; Li et al., 2010; Khushaba et al., 2012). Using extrinsic hand muscles, pattern recognition has been used to effectively classify functional hand-grasp patterns (Li et al., 2010) and even individual finger movements (Tenore et al., 2007; Khushaba et al., 2012), although these previous studies were focused on restoring hand function in trans radial amputees. Partial-hand amputees generally still have an intact, functional wrist, and the muscles controlling wrist motion are also located in the forearm, some superficially. EMG from wrist movements thus degrades the accuracy of classifiers that rely only on extrinsic muscle EMG. Some studies have addressed this by switching between models based on the current wrist position (Pan et al., 2014), but this requires either a parallel classifier or additional instrumentation on the affected hand in order to determine the wrist position in real-time. Adding classifier training data collected from various wrist positions lessens, but does not eliminate, this effect (Adewuyi et al., 2015). Our study expanded upon the results of previous work (Earley et al., 2014) by investigating the interaction and simple main effects of EMG electrode placement and inclusion of EMG data collected during different types of wrist movements; these results were used to set the parameters of the real-time controller.

Several techniques may improve classification accuracy, such as providing more data by extracting EMG features from a longer window (Smith et al., 2011). However, longer data windows result in an increased delay between the onset of the intended movement and intent recognition, which has a negative effect on real-time performance (Farrell and Weir, 2007). Accuracy can also be improved by making fewer classes available to the classifier. Although this generally decreases the complexity of the classification problem, it may not be as functionally useful for the patient. However, because current myoelectric partial-hand prostheses cannot switch between grasps without first being fully-opened, we subdivided grasping tasks into two modes: *grasp selection*, which we define as the initial choosing of a prehensile grasp, and *grasp maintenance*, which only has the option of opening or closing the hand.

**Figure 1:**
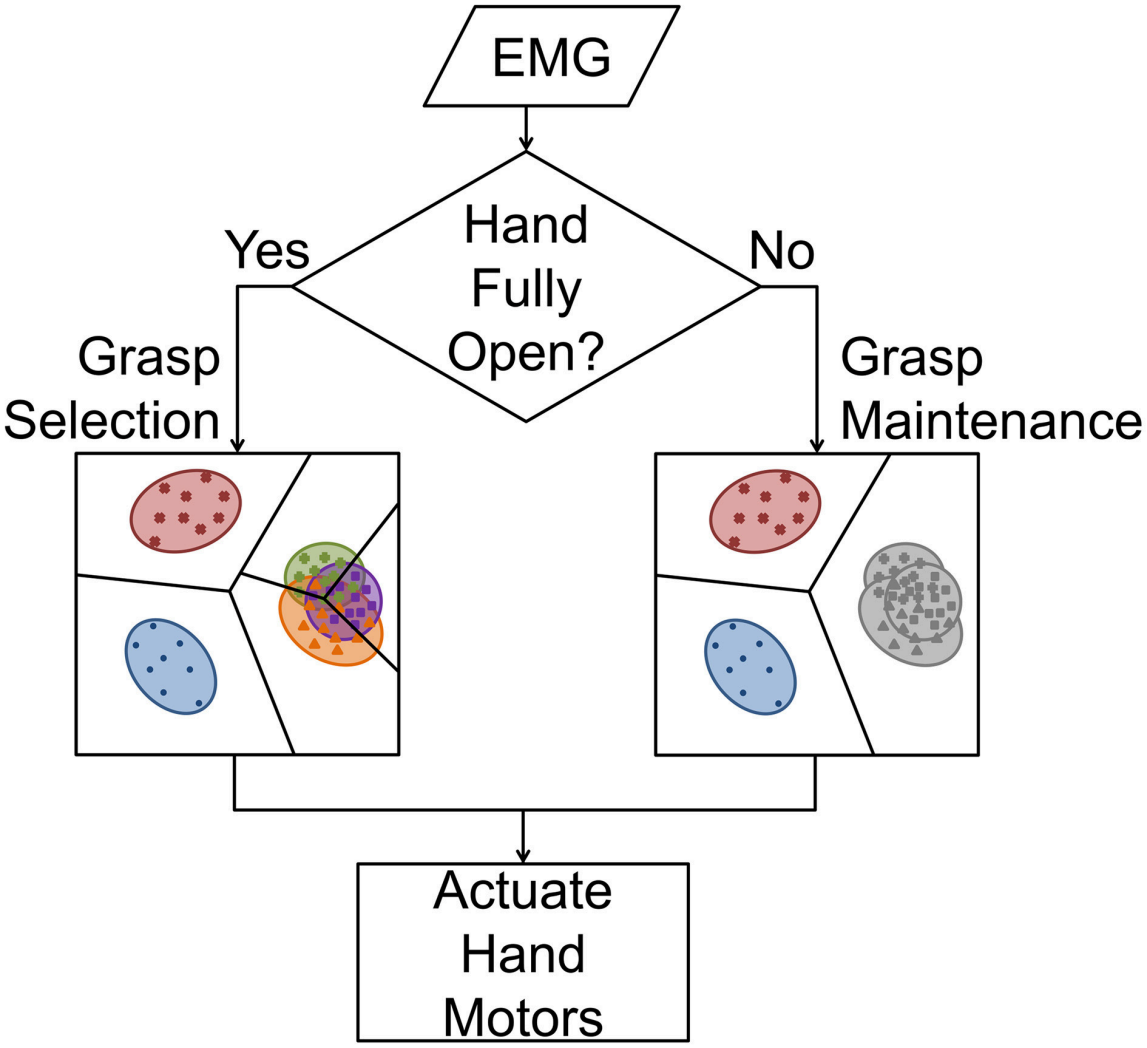
**Prosthetic hand control flowchart**. When the hand is fully open, the LDA classifier must select between *no movement, hand open*, and one of *N* hand-grasps (grasp selection). The use of a longer feature extraction window reduces class variance and, therefore, increases inter-class separability. If a grasp has been selected and the hand is not fully open, all *N* hand-grasps are mapped to *hand close*, and the classifier selects between these three classes (grasp maintenance). Because all hand-grasps are mapped to a common class, a shorter feature extraction window can be used to reduce system delay.

During *grasp selection*, the classifier must select from all possible classes consisting of *N* grasps, *hand open*, and *no movement*, or a total of *N* + 2 classes. After the grasp is selected, the prosthesis changes into *grasp maintenance* mode, and all hand-grasps are mapped to a general *hand close* class. This results in a classifier that makes one of only three possible decisions: *no movement, hand open*, or *hand close* (see Figure 1). In this study, we developed a system that takes advantage of this unique control scheme by dynamically changing the data window length according to the number of available grasps. This *dual window* classifier utilizes a longer data window when more classes are available (*grasp selection*) to improve prediction accuracy, and switches down to a shorter data window when fewer are available (*grasp maintenance*) in order to minimize delay. The lengths of these windows were selected based on the analyses of the offline experiment. In tests evaluating real-time control of a virtual prosthesis, a *dual window* classifier was compared to a classifier that used a consistent data window length for any number of available grasps. Each classifier was tested with different classification techniques to determine the optimal control strategy.

The objective of this study was to develop and evaluate a pattern recognition control system specifically for the partial-hand amputee population. This system was designed to restore a large number of hand-grasp patterns to the user and to accommodate residual wrist movements. In offline trials, we tested the hypothesis that training a classifier with (1) EMG data from both extrinsic and intrinsic hand muscles, (2) EMG data collected with the wrist in multiple combinations of static wrist positions and dynamic wrist motions, (3) a longer EMG data window length, or (4) fewer available grasps would yield the lowest offline classifier error rates. We also tested the hypothesis that, for real-time prosthesis control, using (1) a *dual window* classifier or (2) a majority voting or induced classification delay technique would result in the highest performance.

## Methods

### Experimental protocol

**Figure 2:**
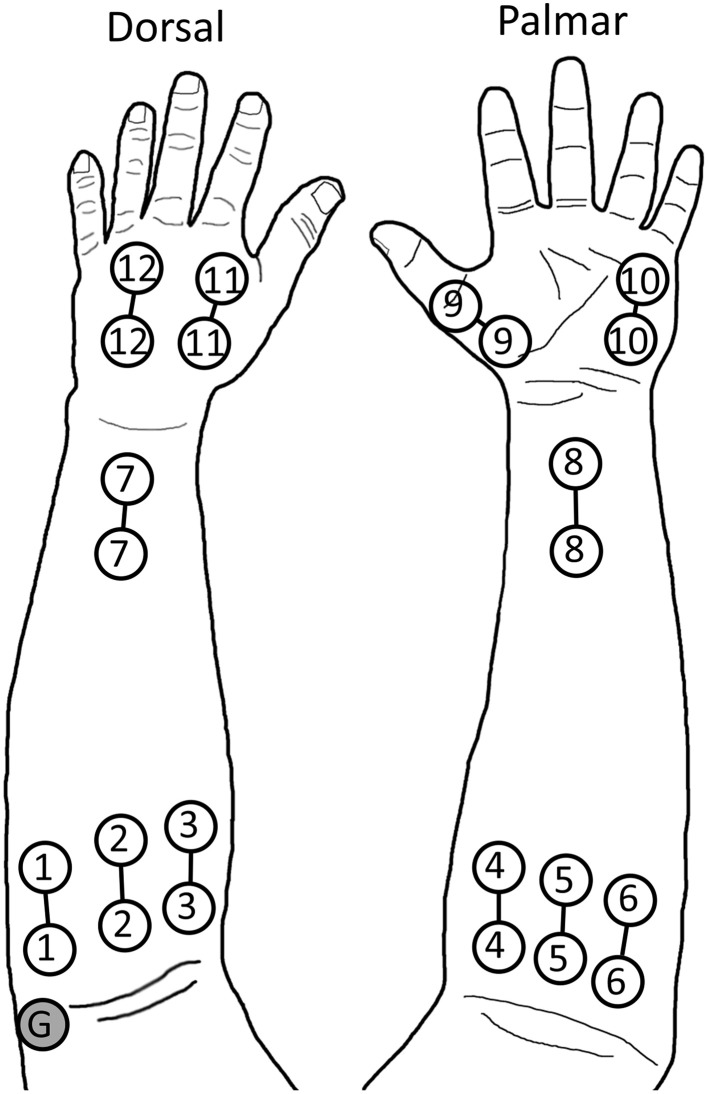
**Electrode placement during experiments**. Electrodes 1–8 record extrinsic muscle EMG, while electrodes 9–12 record intrinsic muscle EMG. The ground (G) is placed on the lateral epicondyle of the humerus. The hand and forearm were then wrapped with an elastic cohesive bandage. © 2014 IEEE. Reprinted, with permission, from Earley et al. (2014).

Seventeen non-amputee subjects and two partial-hand amputee subjects were recruited for this study, which was approved by the Northwestern University Institutional Review Board. Both partial-hand amputee subjects had trauma-related amputations at the metacarpophalangeal joint of digit I. Informed written consent was obtained from all subjects prior to beginning the study. The study comprised two experiments; 14 non-amputee subjects participated in an offline experiment, and nine non-amputee subjects and both partial-hand amputee subjects participated in a real-time experiment. For both experiments, 12 self-adhesive bipolar surface Ag/AgCl EMG electrode pairs were placed on the arm and hand of each subject. Eight electrode pairs covered the extrinsic hand muscles: six placed evenly around the circumference of the proximal forearm, and two on the anterior and posterior sides of the distal forearm. Four electrode pairs were placed directly on the hand to record intrinsic hand muscle EMG: one on the thenar eminence; one on the hypothenar eminence; and two on the dorsal side of the hand (one between the first and second metacarpals, and one between the third and fourth metacarpals). Bipolar electrode pairs had an inter-electrode distance of 4 cm (Young et al., 2012). A reference electrode was placed over the lateral epicondyle of the humerus or olecranon (see Figure 2). The hand and the forearm were lightly wrapped with an elastic, cohesive bandage to prevent electrode displacement.

**Figure 3:**
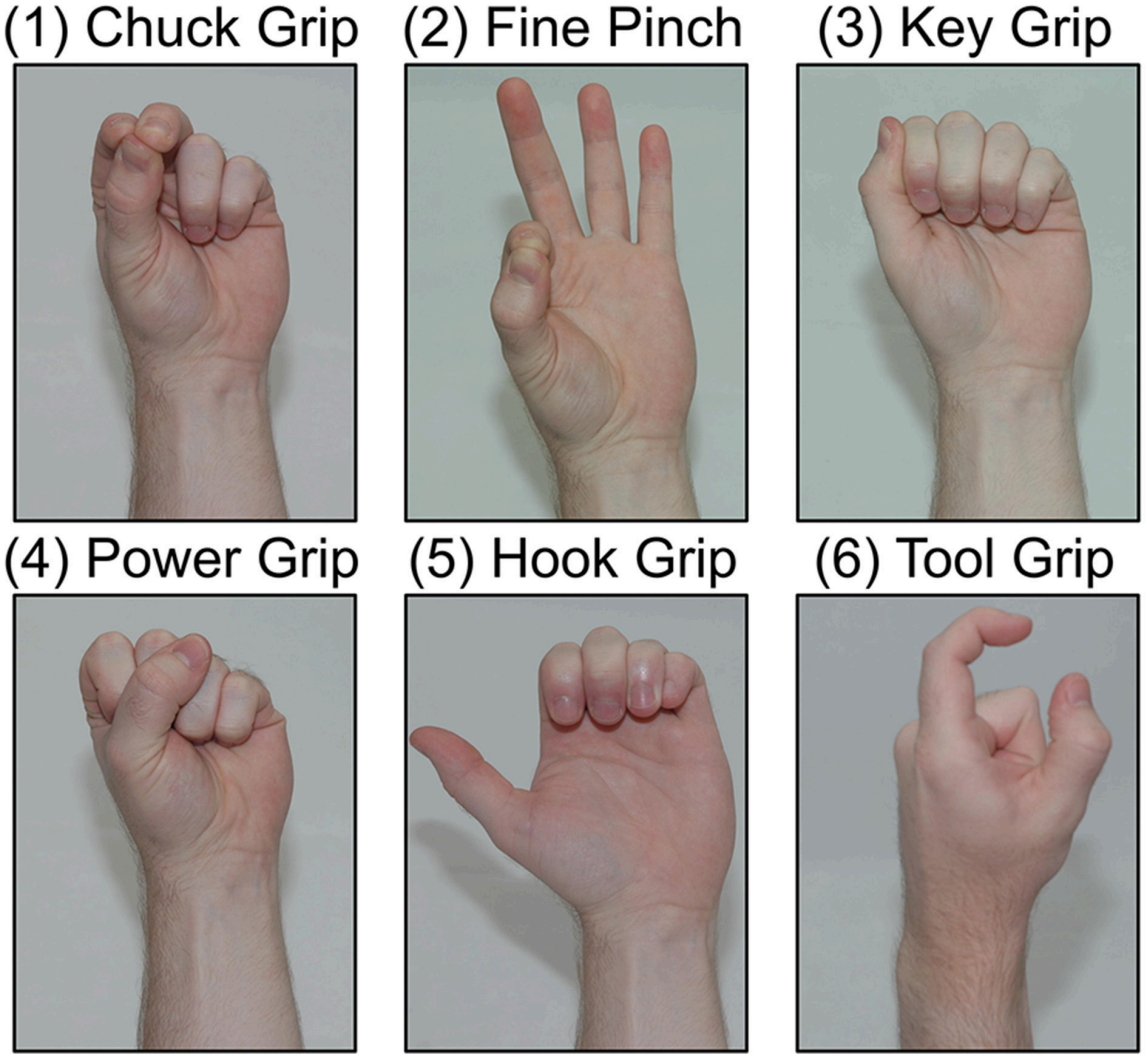
**Tested hand-grasp patterns**. Grasps are ordered from most to least used to perform activities of daily living (ADLs): chuck grip, fine pinch, key grip, power grip, hook grip, tool grip.

Up to six grasps were performed during experiments. These grasps, listed in order of most to least often used for activities of daily living (ADLs), based partially on the results of (Bullock et al., 2013), were chuck grip, fine pinch, key grip, power grip, hook grip (as used to hold a briefcase handle), and tool grip (as used to squeeze the trigger of a power drill; see Figure 3). Subjects also performed *no movement* and *hand open* postures, for a total of eight classes.

### Signal processing

Signal acquisition was guided by custom computer software (Kuiken et al., 2009), which sampled EMG signals at 1000 Hz with a 30–350 Hz band-pass filter using TI ADS1298 biosignal amplifier chips. Four time-domain and six auto-regressive features were extracted from these data (Tkach et al., 2010); all feature extractions were performed with a 25 ms frame increment. A linear discriminant analysis (LDA) classification algorithm was used for pattern recognition, taking advantage of both its relatively simple and efficient calculations, and its equivalent performance to other classification approaches (Hargrove et al., 2007, 2010).

### Offline experiment

**Figure 4:**
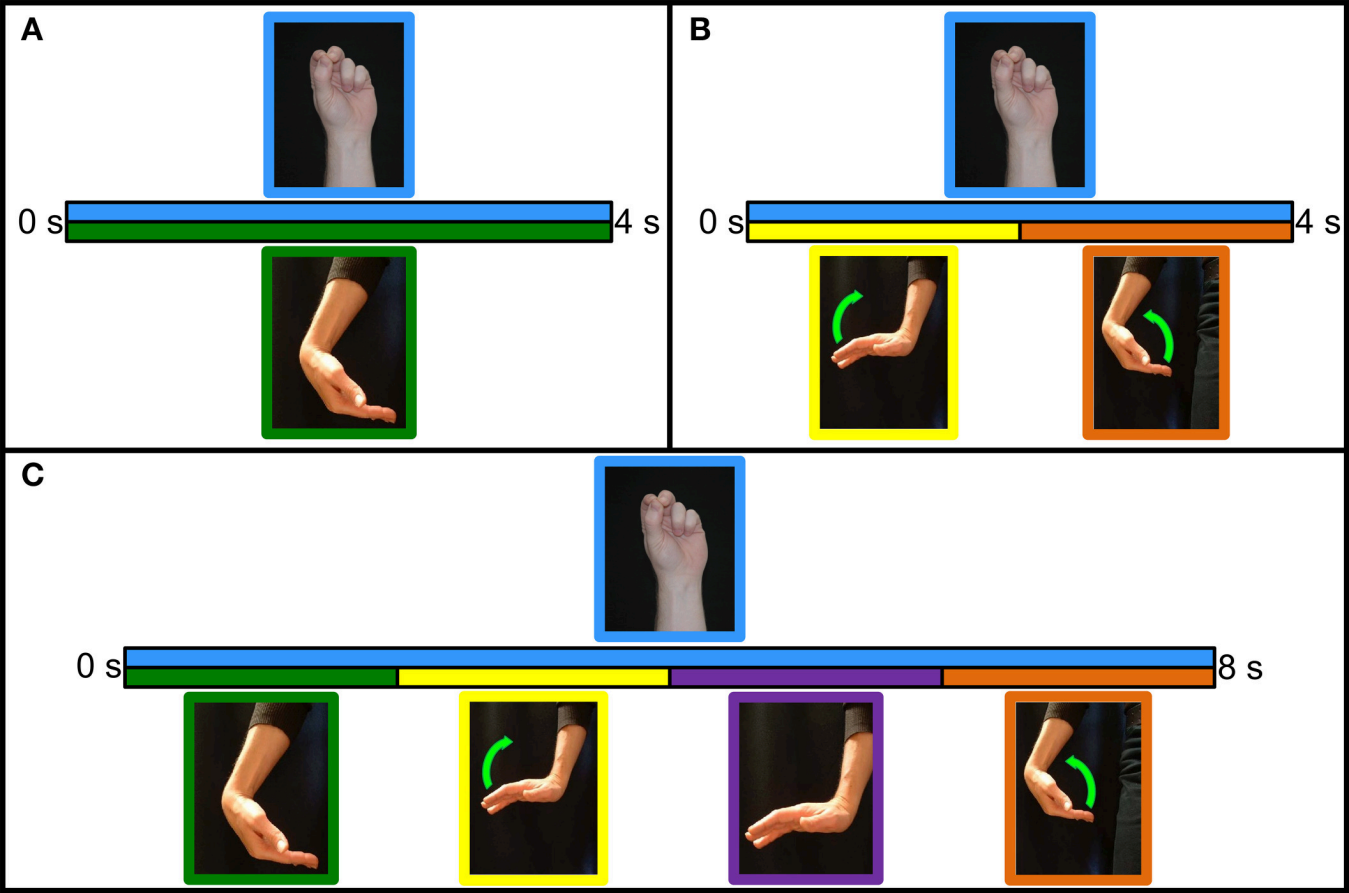
**Classifier training protocols. (A)** *Variable wrist position* protocol. Grasp is initiated and maintained for 4 s in the desired wrist position. **(B)** *Dynamic wrist motion* protocol. Grasp is initiated with the wrist in the starting position. With the grasp maintained for the entire duration, the wrist moves to the opposite position for 2 s before moving back to the starting position for 2 s. **(C)** *Hybrid wrist motion* protocol. Grasp is initiated and maintained in the starting position. Wrist held in the starting position for 2 s, moves to the opposite position for 2 s, held in the opposite position for 2 s, and moves back to the starting position for 2 s.

An offline experiment was performed to determine the best possible method to train the control system for performing hand-grasp selection and to familiarize subjects with pattern recognition prosthesis control prior to the real-time experiment. Data were collected using the protocol from a previous study (Earley et al., 2014), and data from this previous study were combined with these new data to perform statistical analysis on interaction and simple main effects, which influenced the development of the real-time algorithms detailed in the next section. EMG data were collected under 10 conditions. Subjects first held the wrist in a neutral position and performed the eight hand postures (six grasps, *hand open*, and *no movement*) for 4 s each. These postures were then performed with the wrist statically held in flexion, extension, radial deviation, ulnar deviation, pronation, and supination. Each hand posture was performed 4 times in each wrist position. Data collected during these seven static wrist conditions were combined into the *variable wrist position* data set (see Figure 4A). Subjects were then asked to perform hand postures four times while moving the wrist from flexion to extension, before returning to flexion, and four times while moving the wrist from extension to flexion, and back to extension. Wrist movements spanned 2 s in each direction, for a total of 4 s. This procedure was repeated for wrist radial and ulnar deviation, and pronation and supination; data collected during these three dynamic conditions were combined into the *dynamic wrist movement* data set (see Figure 4B). Analyses were performed using two-fold cross-validation. Classification performance was quantified by classification error, which is the percentage of incorrect classifications.

Two statistical analyses were performed, both with subjects as a random variable: an ANOVA with electrode placement and training method as factors, and an ANOVA with window length and available grasps as factors; main effect and interaction effect terms were included in each model. If the interaction was not found to be significant, the analysis was rerun with a reduced model consisting only of main effects. When interaction was found to be significant, a subsequent analysis was performed to determine the simple main effects. Pairwise comparisons were made using Bonferroni correction factors, and significance levels were set at α = 0.05. Analyses were split into two categories: *grasp selection* and *grasp maintenance* performance. *Grasp selection* was only tested on *various wrist positions* data—from an application perspective, this equates to prepositioning the wrist prior to making a hand-grasp selection. *Grasp maintenance* was tested on both *various wrist positions* and *dynamic wrist motions* data—this equates to the user being able to move their wrist freely, after grasp selection, as they maintain the selected grasp.

EMG training data were obtained from (1) extrinsic muscles, (2) intrinsic muscles, or (3) extrinsic and intrinsic muscles. Classifiers were trained with one of four sets of training data: (1) the wrist only in the neutral position, (2) the wrist in seven variable wrist positions, (3) the wrist moving in each of the three degrees of freedom (dynamic wrist movement), and (4) all static and dynamic wrist data. All possible combinations of electrode position and wrist position yielded a total of 12 conditions. *Grasp selection* analyses were performed with a 500 ms EMG feature extraction window (Smith et al., 2011), trained on all data in one cross-validation fold, and tested against the first 600 ms of each static trial included in the other fold, effectively evaluating the performance of the classifier on only transient EMG generated by the onset of grasp selection (Hudgins et al., 1993). *Grasp maintenance* analyses were performed with a 200 ms EMG feature extraction window, and tested against all but the first 300 ms of each included data collection.

Analyses of window length and available grasps were performed on tests run with the classifiers trained with both dynamic and static wrist movements and with both extrinsic and intrinsic EMG channels. Pattern recognition classifier performance was evaluated for 100, 200, 300, 400, and 500 ms feature extraction window lengths; and for *N* (2, 4, or 6) grasps available to the classifier, where grasps were the *N* most useful hand-grasps for performing ADLs (see Figure 3). In order to ensure adequate data to train a classifier for grasp selection analyses with feature extraction window length *L*, the first *L*+100 ms of each data collection were used, capturing the transient EMG from each grasp initiation. The remaining durations of data collections were used for grasp maintenance analyses for all window lengths.

### Real-time experiment

A second experiment was performed to evaluate real-time prosthesis control, including the selection of an intended grasp in different wrist positions and the ability to re-attempt a failed grasp selection. During this experiment, subjects used extrinsic and intrinsic muscle EMG to control a virtual reality (VR) hand simulating a multi-articulate hand prosthesis with proportional control (Lock et al., 2005) and a 500 ms velocity ramp (Simon et al., 2011). Prior to the start of the experiment, training data were collected for *no movement* and *hand open* postures, and for chuck, fine pinch, key, and power grips. First, subjects held hand postures for 4 s with the wrist in a neutral position; this was repeated twice per posture. Subjects were then asked to perform and maintain hand postures while moving the wrist along a specific trajectory lasting 8 s. Starting with the wrist flexed, subjects initiated and held a hand posture for 2 s, moved from wrist flexion to extension over the next 2 s, held in extension for 2 s, and moved back to wrist flexion over the last 2 s. Data collection was performed similarly for wrist extension, radial and ulnar deviation, and pronation and supination (see Figure 4C). These data were used to train classifiers controlling the VR hand. Subjects were given a few minutes to practice controlling the VR hand with a 250 ms static window classifier before starting the experiment.

During this experiment, subjects started with the wrist in a pseudo-randomly selected position. The pseudo-random wrist position selection was structured so that every combination of 7 wrist position and 4 grasps was tested once, yielding 28 trials per test. At the start of each trial, the VR hand was obscured and a window informed the subject of the intended wrist position; the subject had 2 s to move their wrist to the required position. The VR hand then became visible, the target grasp was displayed, and control of the hand was relinquished to the subject. Subjects had 15 s to fully close the VR hand in the indicated grasp to complete the trial successfully. If subjects were unable to complete the trial within 15 s, it was marked as timed out and they continued to the next trial.

We evaluated different windowing methods and classification techniques. The two windowing methods evaluated were (1) static window (250 ms) and (2) dual window (500 ms during *grasp selection*, 200 ms during *grasp maintenance*). Two-hundred and fifty milliseconds was selected for the static window due to its pervasive use in current prosthesis control applications. Five-hundred and two-hundred milliseconds were selected for the dual windows based on results of the offline experiment, providing the lowest possible classification error and the fastest possible response time without a significant increase in classification error, respectively. The dual window classifier was signaled to switch between *grasp selection* and *grasp maintenance* modes based on the position of the virtual hand; a fully-open hand indicated *grasp selection* mode, and a partially- or fully-closed hand indicated *grasp maintenance*. For each windowing method, three classification techniques were evaluated: (1) unmodified LDA, (2) classification delay, and (3) majority voting. For (1) and (2), a single classification being made from the current data window, while for (3), recent predictions were tallied; the majority voting window was the same as the feature extraction window length (Englehart and Hudgins, 2003). An additional constraint was implemented requiring a minimum of 50% of the possible votes in order to make a classification; otherwise, the system would default to *no movement*. This prevented frequent class switching due to unintentional EMG activity or other non-volitional causes.

Classification delay (2) was implemented by freezing motor control of the hand whenever the hand was moved to the fully-open position. This freeze persisted until consecutive grasp selection classifications surpassing half the duration of the current window length had been issued; therefore, motor control would be frozen for a minimum of 125 ms during static window tests and 250 ms during dual window tests. Any classifications of *hand open* or *no movement* reset this timer. This method was implemented to prevent unintentional classifications while the subjects' hands moved from fully-open to the desired grasp.

Subjects performed the experiment described above using one of the six windowing/classification combinations in randomized order. Four quantitative performance metrics were used to evaluate real-time performance. Timeout rate was the percentage of the 28 trials that were not completed successfully within the 15 s limit. Selection time was the elapsed time between the start of the trial (when the VR hand was revealed to the subject) and when the correct grasp was selected; likewise, completion time was the elapsed time between this initial grasp selection and the full closure of the VR hand. Selection attempts were the number of times subjects made a grasp selection with the hand fully-open; one selection attempt indicated that a subject achieved the correct grasp successfully on the first try, whereas higher values indicated repeated attempts. Data from incomplete trials were not included in calculations of average selection time, completion time, or selection attempts. Subjects were also asked to rate each classifier on a scale from 1 to 10, with the practice classifier scoring a 5 to ensure a similar baseline across subjects.

A three-way ANOVA was used to analyze the results, with windowing method and classification technique as fixed factors and subjects as a random factor. To account for non-normal data, a Box–Cox transformation was used (Box and Cox, 1964). Pairwise comparisons were made using a Bonferroni correction factor, and the significance level was set at α = 0.05.

## Results

### Offline experiment

**Figure 5:**
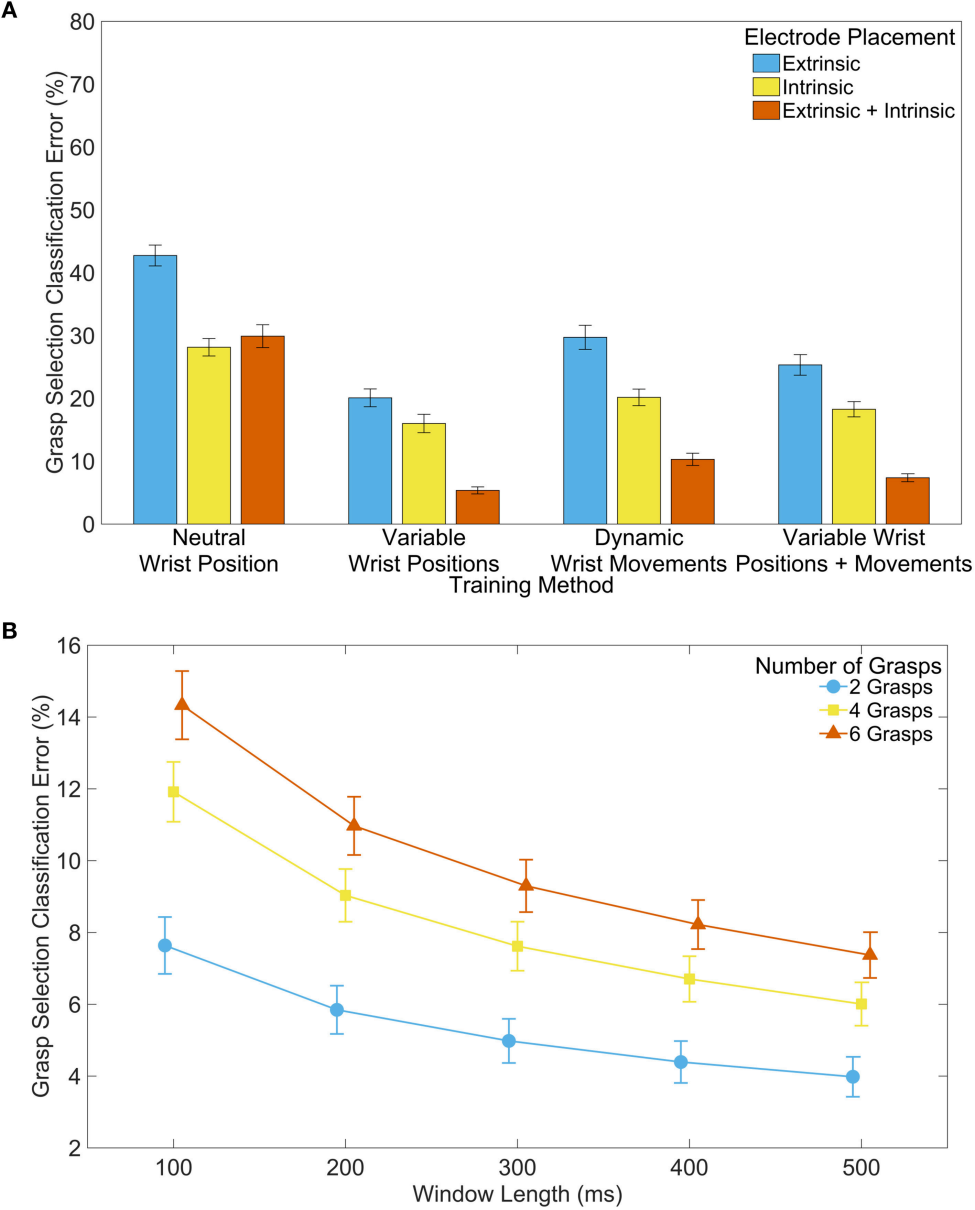
**Classifier performance during grasp selection**. **(A)** Effect of training method and electrode placement on classification error. Analysis performed with 500 ms feature extraction window and 6 grasps available to the classifier. Error bars represent standard error. **(B)** Effect of window length and number of grasps available to the classifier on classification error. Analysis performed with extrinsic and intrinsic muscle EMG, and with variable wrist positions and dynamic wrist movements included in the training data. Error bars represent standard error. Error bars represent standard error.

Compared to training in only the neutral wrist position, classification error was significantly reduced through training with grasp selection data collected (1) from different static wrist positions, (2) during dynamic wrist movements, or (3) with a combination of these two methods (*p* < 0.001 for all simple effects). Training with different static wrist positions performed better than training with dynamic wrist movements (simple effects: *p* < 0.05); these findings were expected because grasp selection was evaluated with a static wrist, and training methods containing these data most closely match the test data (see Figure 5A).

The data show significant interaction effects between the training method and electrode placement (*p* < 0.001), indicating the importance of intrinsic EMG signals for instances where wrist information was not included in the training data. We found a significant decrease in classification error training with only intrinsic EMG signals, compared with only extrinsic EMG signals (simple effects: *p* < 0.001), and training with a combination of extrinsic and intrinsic muscle EMG data was significantly better than either extrinsic or intrinsic alone (simple effects: *p* < 0.05) with the exception of the comparison between extrinsic and intrinsic muscle EMG and only intrinsic muscle EMG when trained with only in the neutral wrist position (*p* > 0.99). Thus, including both extrinsic and intrinsic EMG signals in the training data generally provided the lowest classification error (see Figure 5A).

Significant interaction effects between the window length and the number of grasps available to the classifier (*p* < 0.001) illustrate the increasing importance of a longer EMG window for classification accuracy as more grasps become available. Increasing the feature extraction window length reduced classification error (see Figure 5B), supporting the results of previous studies (Smith et al., 2011). Furthermore, using classifiers with fewer available grasps reduced error significantly, as hypothesized. The interaction between window length and number of available grasps can be clearly seen in Table S3, where the differences between means are larger when more grasps are available and smaller when fewer grasps are available.

**Figure 6:**
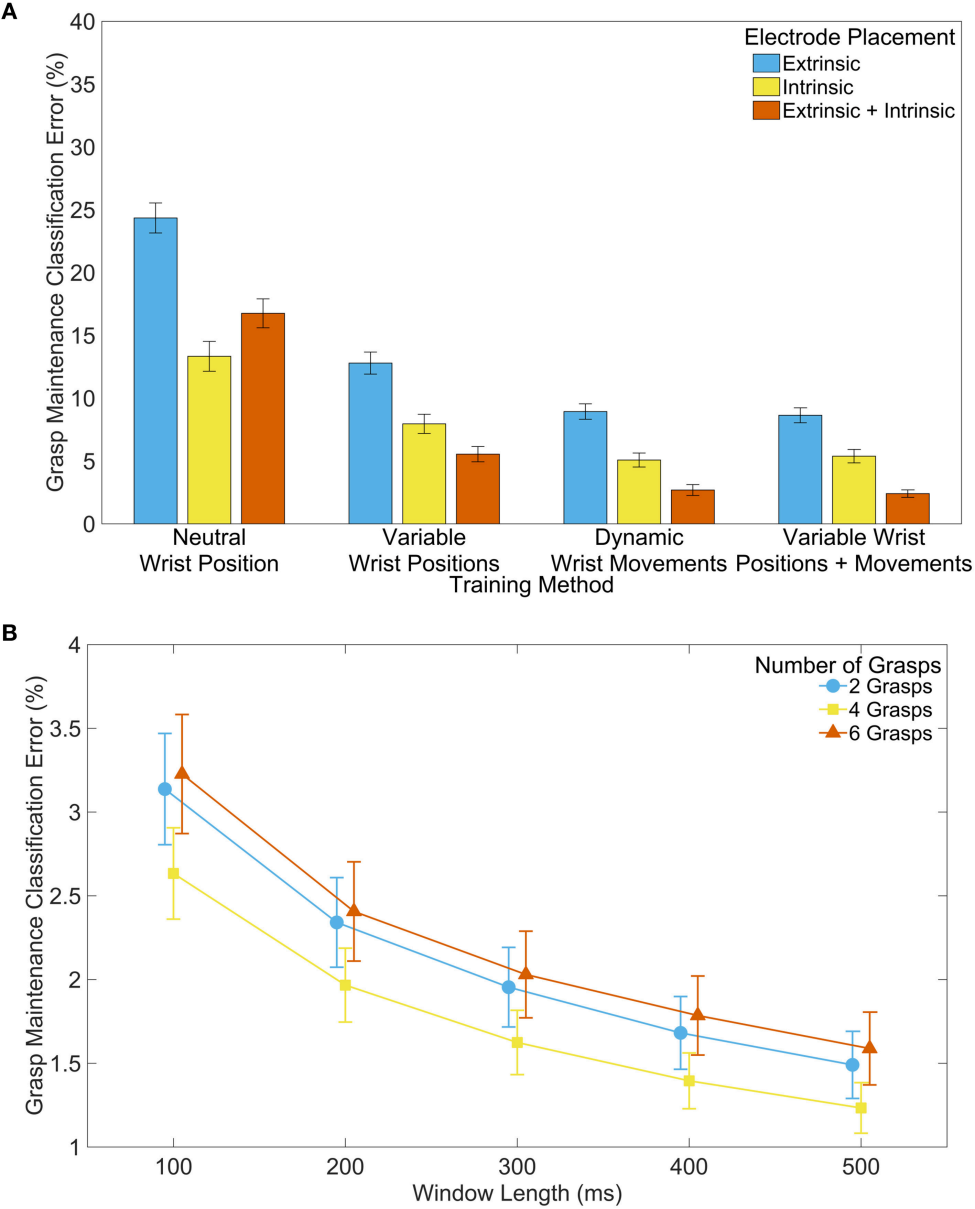
**Classifier performance during grasp maintenance**. **(A)** Effect of training method and electrode placement on classification error. Analysis performed with 200 ms feature extraction window and 6 grasps available to the classifier. **(B)** Effect of window length and number of grasps available to the classifier on classification error. Analysis performed with extrinsic and intrinsic muscle EMG, and with variable wrist positions and dynamic wrist movements included in the training data. Error bars represent standard error.

The trends for *grasp maintenance* were similar to those seen during *grasp selection*. The inclusion of static and dynamic wrist information yielded the lowest error (see Figure 6A). There were also significant differences between using only extrinsic, only intrinsic, and all muscle EMG; using all muscle EMG provided the lowest classification error. Increased feature extraction window lengths again showed reduced classification error. However, trials with two and six available grasps were not significantly different for any window length (see Figure 6B).

Pairwise comparison tables for the simple main effects analyses have been provided in the Supplementary Material. Table S1 shows simple effect pairwise comparisons between training methods, for each electrode placement. Table S2 shows simple effects between electrode placements for different training methods. Table S3 depicts simple effects of window length for all levels of available grasps, and Table S4 depicts simple effects of available grasps for all levels of window length.

### Real-time experiment

**Figure 7:**
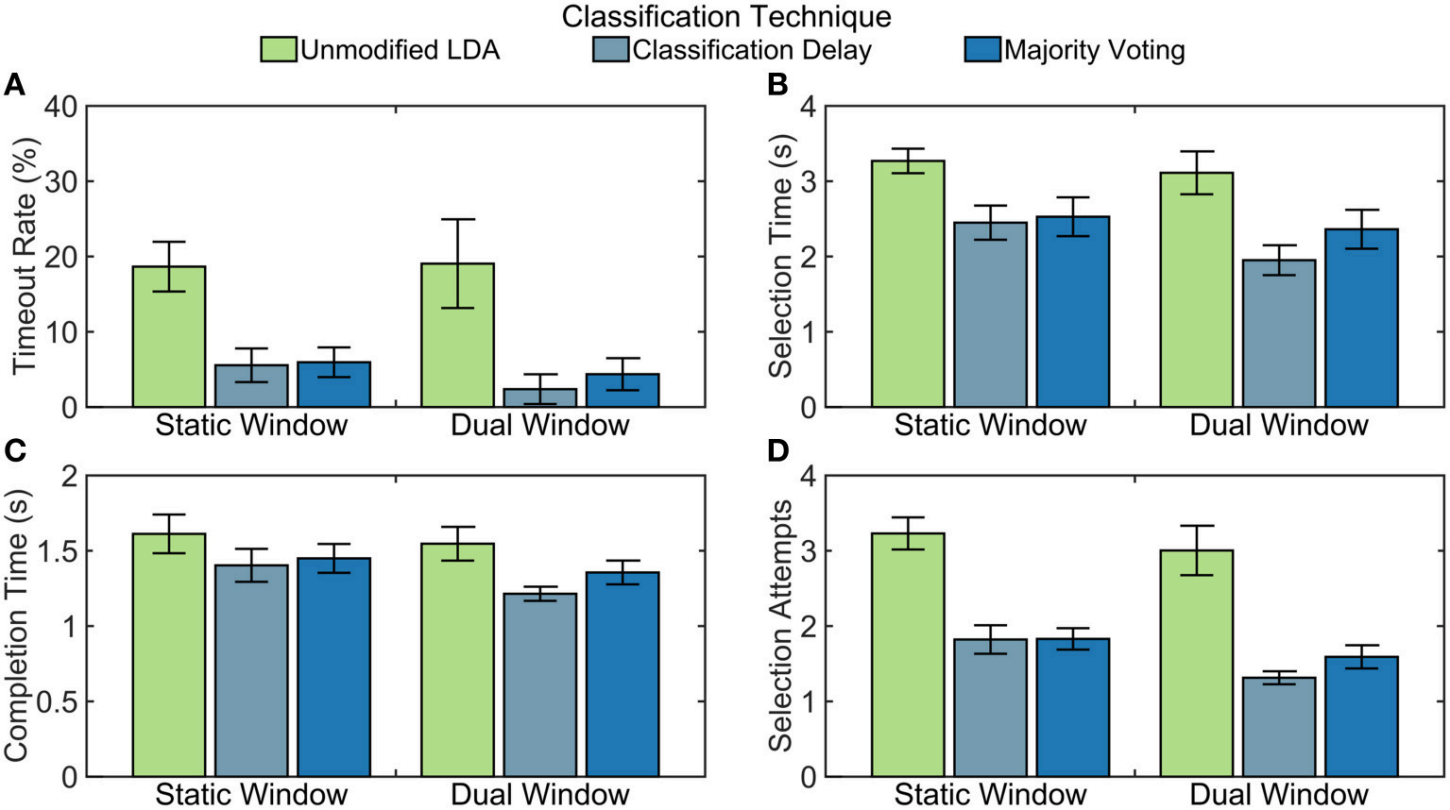
**Non-amputee real-time performance of tested algorithms**. Quantitative results from real-time experiments with non-amputee subjects. Error bars represent standard error. Calculations for **(B–D)** do not include timed-out trials. **(A)** Timeout rate is calculated as the percentage of the 28 trials that were not completed within 15 s. **(B)** Selection time is calculated as the elapsed time between when the trial started and when the correct grasp was selected. **(C)** Completion time is calculated as the elapsed time between when the correct grasp was selected and when the grasp was fully closed. **(D)** Selection attempts are the number of times a grasp was selected. Every time the hand transitioned from fully-open to partially-closed, this counter was incremented.

Significant interaction was found between windowing methods and classification technique for selection attempts (*p* < 0.05), but not for any other metric (*p* > 0.276). The test timeout rate was significantly lower when either the classification delay (15%) or the majority voting (14%) techniques were used, compared to an unmodified LDA classifier (*p* < 0.001). The effects of classification delay or majority voting were not significantly different. The choice of windowing method did not have a significant effect on timeout rate (*p* = 0.199). Selection time was lower when the majority voting (990 ms, *p* < 0.01) or classification delay (750 ms, *p* < 0.001) techniques were used compared to the unmodified classifier. Completion time improved with a classification delay (270 ms, *p* < 0.001), but the improvement in completion time with majority voting was not significant (*p* = 0.065). The dual window classifier significantly improved both selection (270 ms) and completion time (120 ms, *p* < 0.05). When subjects used an unmodified LDA or a majority voting LDA classifier, the windowing methods did not have a significant effect on the number of selection attempts (*p* = 0.233 and *p* = 0.087, respectively); however, when using the classification delay technique, using the dual window classifier resulted in significantly fewer selection attempts (*p* < 0.01). For both windowing methods, using either the classification delay or majority voting technique resulted in the fewer attempts than using the unmodified LDA (simple effects: *p* < 0.001), but were not significantly different themselves (simple effects: *p* > 0.104; see Figure 7).

**Figure 8:**
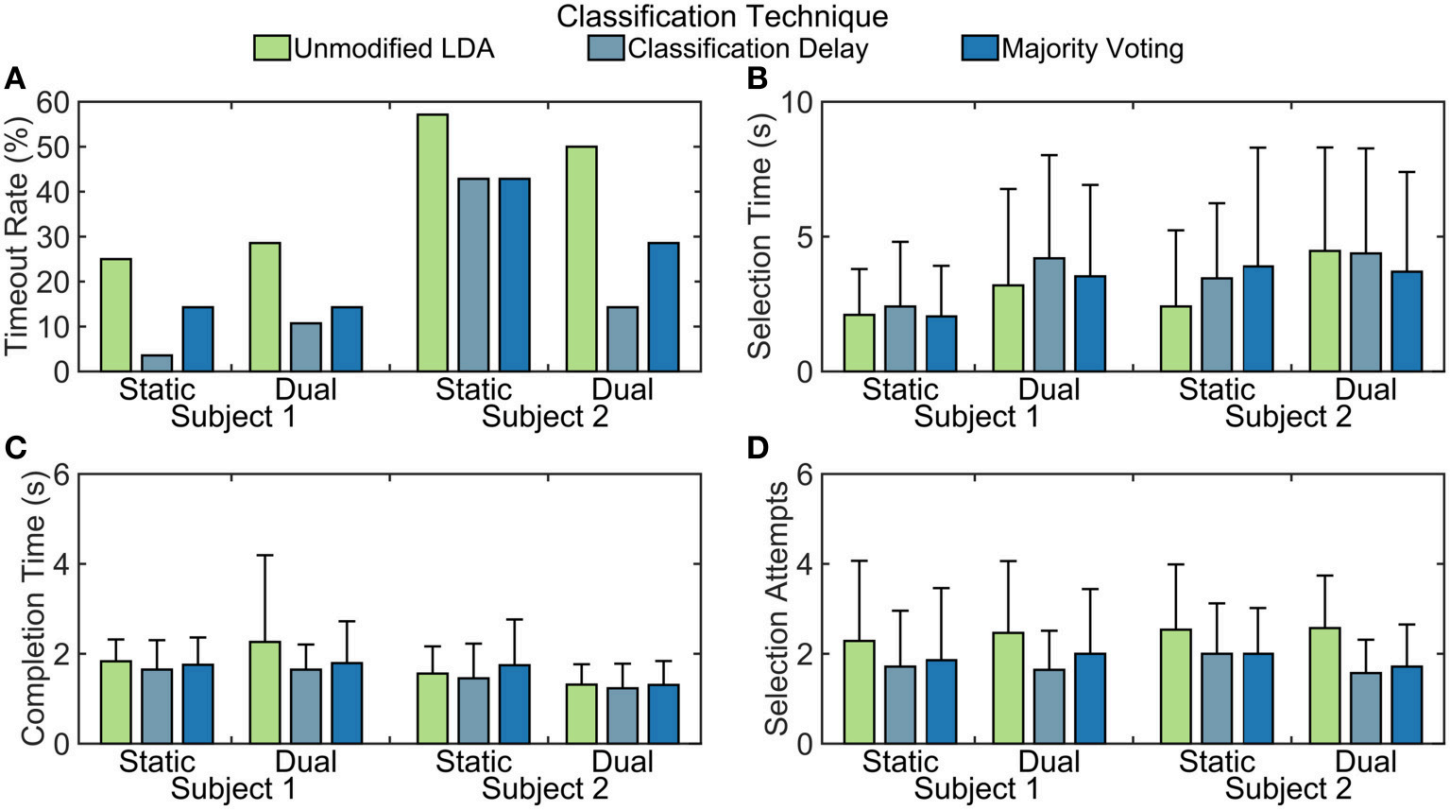
**Partial-hand amputee real-time performance of tested algorithms**. Quantitative results from real-time experiments with partial-hand amputee subjects. Error bars represent standard deviation from 28 trials. Calculations for **(B–D)** do not include timed-out trials. **(A)** Timeout rate is calculated as the percentage of the 28 trials that were not completed within 15 s. **(B)** Selection time is calculated as the elapsed time between when the trial started and when the correct grasp was selected. **(C)** Completion time is calculated as the elapsed time between when the correct grasp was selected and when the grasp was fully closed. **(D)** Selection attempts are the number of times a grasp was selected. Every time the hand transitioned from fully-open to partially-closed, this counter was incremented.

For both amputee subjects, the classification delay and majority voting techniques resulted in a lower timeout rate than the unmodified LDA, meaning that they were able to complete more tasks (see Figure 8). These two techniques also allowed the amputee subjects to make fewer select attempts on average during trials. Although it appears that the selection time is lowest with the unmodified LDA, this is not unexpected; trials that timed out were not included in calculations for other metrics, resulting in shorter selection and completion times. Subject two had a lower timeout rate with the dual window classifier than with the static window classifier, but other comparisons between windowing methods are inconclusive with only two amputee subjects.

**Figure 9:**
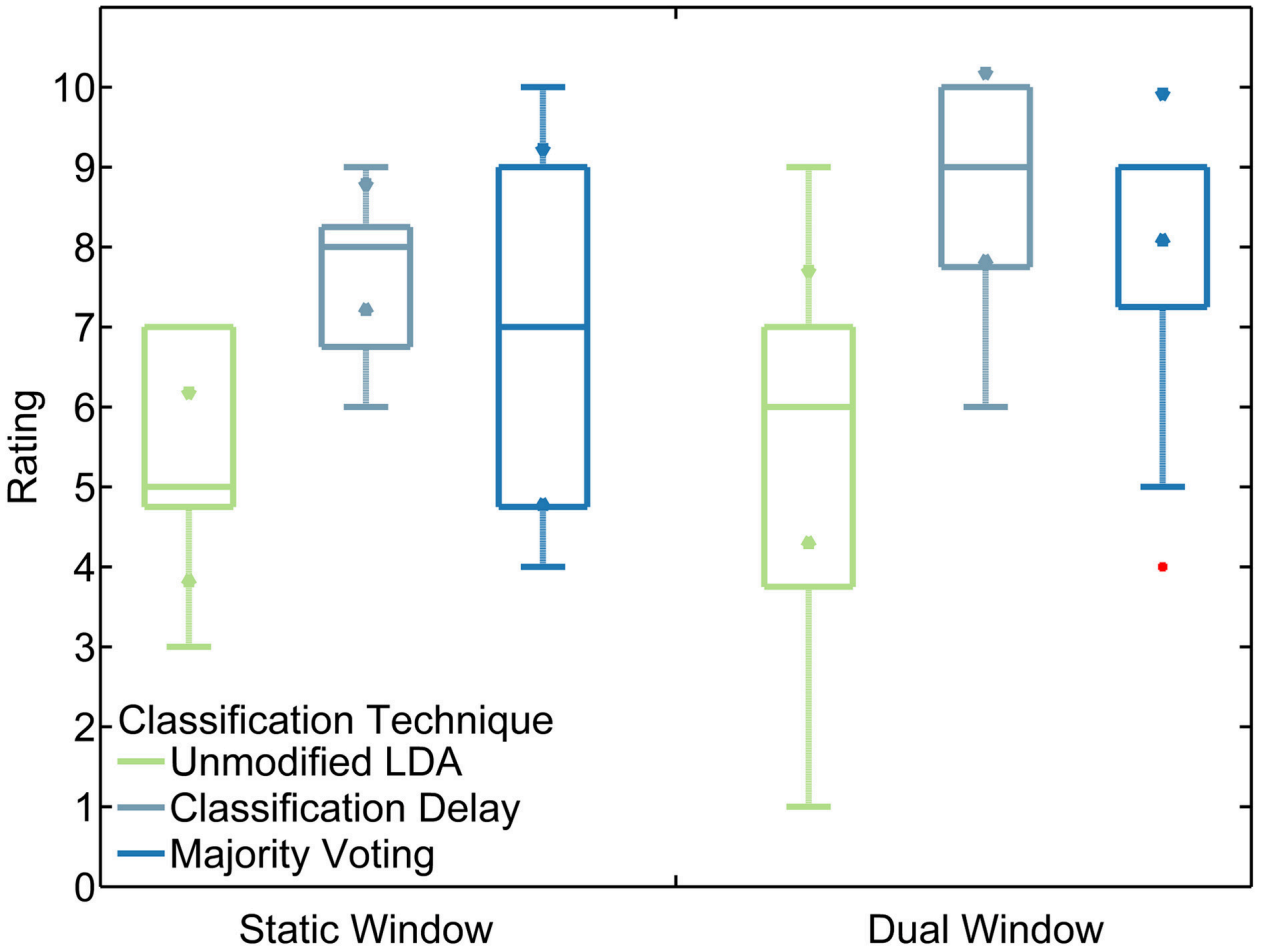
**Non-amputee ratings of tested algorithms**. Boxes represent median and inter-quartile ranges. Triangle markers represent 95% confidence intervals. Red points represent outliers. Rankings were given on a scale from 1 to 10. Subjects were asked to compare all algorithms to the practice algorithm (unmodified LDA, static window), which they were instructed to consider to be rated as 5.

**Figure 10:**
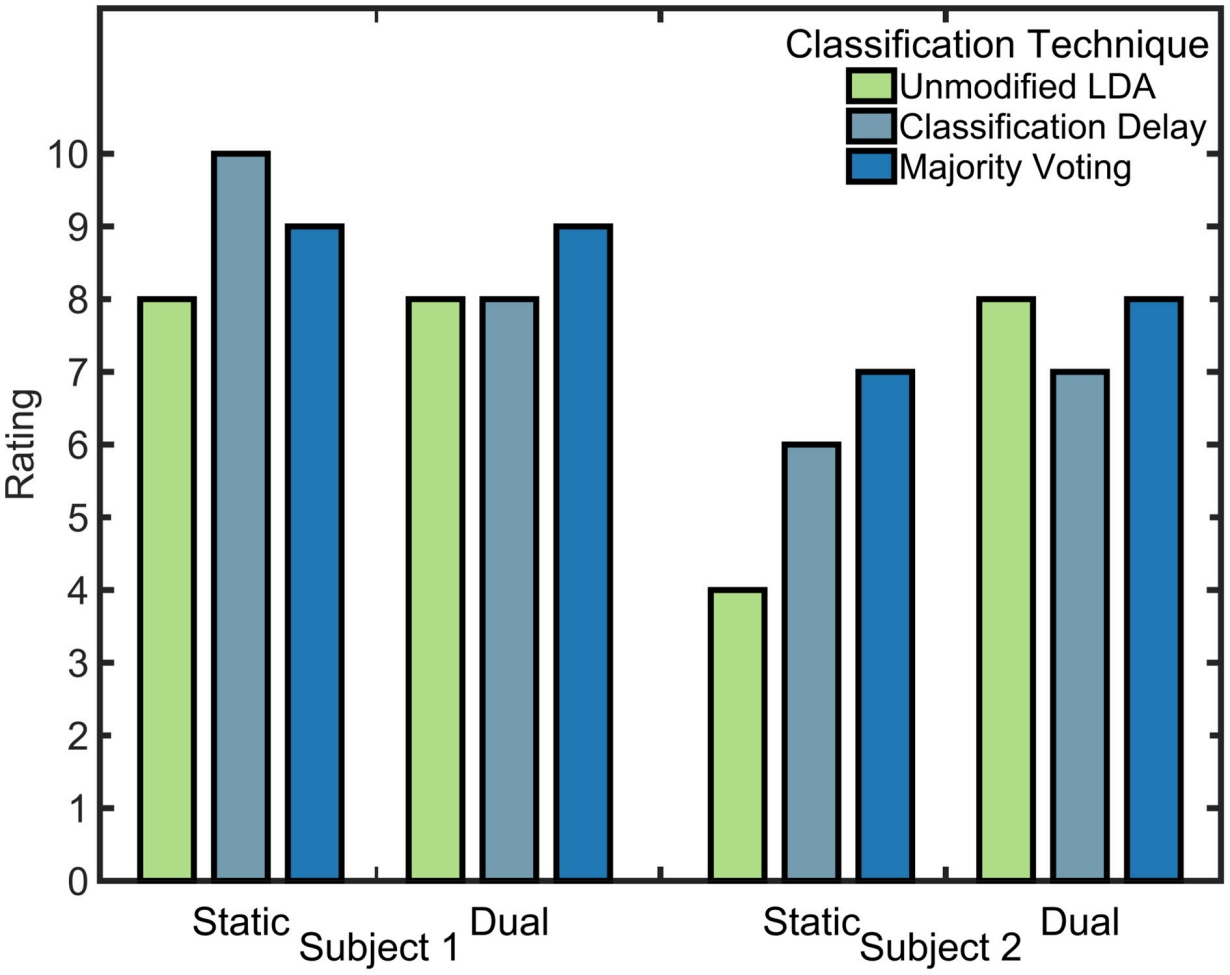
**Partial-hand amputee ratings of tested algorithms**. Rankings were given on a scale from 1 to 10. Subjects were asked to compare all algorithms to the practice algorithm (unmodified LDA, static window), which they were instructed to consider to be rated as 5.

When asked to rate the control schemes on a scale from 1 to 10, in which the static window/unmodified LDA scheme that subjects practiced with before starting the tests was set as a five, subjects typically rated schemes with the classification delay and majority voting techniques higher than the unmodified classifier (*p* < 0.001). Classification delay appeared to be rated higher than majority voting, on average, though not by a statistically significant margin (*p* = 0.098). Subjects also rated schemes with the dual window classifier higher than those with a static-window classifier (*p* < 0.05; see Figure 9). For both amputee subjects, the classification delay and majority voting techniques rated at least as highly as the unmodified LDA, except for subject two rating the unmodified LDA higher than the classification delay technique for the dual window classifier. In addition, subject two consistently rated the dual window classifiers more highly than the static window classifiers (see Figure 10).

## Discussion

Myoelectric partial-hand prostheses only recently became commercially available, thus research on the control of these devices is limited. Improving the control of myoelectric partial-hand prostheses is especially important because partial-hand amputees perceive themselves to be less functional than individuals with higher-level amputations (Davidson, 2004). Controlling a partial-hand prosthesis presents different challenges than controlling a trans radial device, most notably because a partial-hand amputee has an intact, functional wrist (Lang, 2011). However, few have studied the effect of wrist kinematics on control of partial-hand prostheses. Adewuyi et al. found that wrist movements significantly degraded classifier performance, and that this effect could be reduced by including EMG data recorded in variable wrist positions and during wrist movement (Adewuyi et al., 2015). Additionally, that study investigated the effect of including intrinsic muscle EMG. Our study expands upon on this investigation by exploring additional training methods, as well as by increasing the number of hand-grasp patterns available to the classifier from two to six available grasps.

In this paper, we proposed and evaluated innovative pattern recognition control schemes for controlling partial-hand prostheses. In offline classifier evaluations, we showed that longer feature extraction window lengths resulted in lower classification error, agreeing with the results of Smith et al. for trans radial prosthesis control (Smith et al., 2011). Furthermore, we demonstrated that longer windows become more important as more classes are available to the classifier. We also showed that, for hand-grasp selection, training a classifier with EMG only collected in the neutral wrist position resulted in much higher classification error than training with EMG collected with the wrist moving or while held in different static positions. Thus, to allow a user to preposition the wrist prior to selecting a hand-grasp, the classifier must be properly trained by including EMG from different static wrist positions or dynamic wrist movements in the training data.

The deterioration in classification error due to wrist motion was most prevalent while controlling a prosthesis using only extrinsic muscle EMG signals, which are more sensitive to the confounding effects of EMG generated by wrist movement; a prosthesis controlled using only intrinsic muscle EMG performed significantly better (*p* < 0.001). Training with static wrist data yielded the lowest error during grasp selection; however, this classifier was not robust enough to perform optimally during dynamic wrist motions. Consequently, we recommend training classifiers with both variable wrist positions and dynamic wrist motions. This will minimize classification error and ensure high performance during both hand-grasp selection and maintenance. This approach can be further improved by using prosthesis-guided training, during which the user is instructed to move the wrist through its range of motion while following along with the appropriate hand-grasp selection cue (Simon et al., 2012).

Controlling prostheses with a combination of extrinsic and intrinsic EMG signals resulted in significantly lower classification error than when controlling with only extrinsic or only intrinsic muscle EMG. Although intrinsic muscle EMG provides data that are most invariant to the effects of wrist motion, it can be difficult to place electrodes in the socket, and depend greatly on the level of amputation of the user. Extrinsic muscle EMG clearly provides complimentary information that, when combined with intrinsic muscle EMG, lowers classification error. Thus to restore more hand-grasp patterns, extrinsic muscle EMG should be used in conjunction with intrinsic muscle EMG to control partial-hand prostheses whenever possible.

During real-time experiments, the classification problem was broken into two paths. First, for the hand-grasp selection component in the proposed dual-window classifier, we used a longer window length than would generally be tolerable for normal control. After hand-grasp selection, control quickly transitioned to a classifier that interprets a shorter window length for normal operation of opening and closing the grasp. For a prosthetic control system, this mode transition could be determined based on current draw from the motors; while the fingers are opening, a current spike would indicate that the hand is fully open and thus prompt a transition from *grasp maintenance* to *grasp selection*.

Our results show that the implementation of a dual window classifier allowed users to make fewer attempts at selecting an intended grasp before succeeding, and also permitted faster grasp closure times. The implementation of either a classification delay or a majority voting technique also showed significant improvement in every performance metric evaluated; furthermore, users rated these control schemes more highly than unmodified LDA classifiers. This is due primarily to the reduced number of attempts users needed to make to successfully achieve a desired grasp. While controlling the VR hand with unmodified classifiers, users would often accidentally trigger an incorrect grasp while moving the fingers and hand from a rest posture to the desired position. This accidental trigger would then lock the prosthesis into this wrong grasp, and the user would have to fully open the hand to reattempt the desired movement. This would cause an increased selection time, stemming from trials where the user selected the correct grasp on the second attempt or later, and a decreased completion rate, stemming from trials where the user was unable to select the correct grasp despite numerous attempts. Although it seems unintuitive to introduce delays into a prosthetic control system designed for minimal latency, the classification delay and the 50% minimum vote threshold in the majority voting technique both served to deter triggering such accidental classifications by bypassing a portion of the transient EMG and focusing more closely on the steady-state EMG.

Including wrist motion in a pattern recognition classifier is critical to maximizing performance of partial-hand prostheses while still preserving residual wrist movement. Although including these data increases the training time of the prosthesis, it is still a reasonable length for daily use. To improve prosthesis performance, EMG should be collected from intrinsic hand muscles in addition to extrinsic muscles, whenever possible. Furthermore, using a dual window classifier maximized classification accuracy when class selection was most important and reduced system delay when additional accuracy was not necessary or fewer grasps were available. Finally, the introduction of classification delays or majority voting techniques also significantly improved real-time prosthesis control and were generally preferred by users. These techniques form the basis for developing a control system for a partial-hand prosthesis that preserves the function of the wrist. The control systems proposed here are simple and can easily be implemented with current devices already available on the market.

The major limitation of this study is that experiments with partial-hand amputee subjects were not performed with a physical prosthesis. It is possible that the weight or configuration of a prosthetic device could affect amputee performance and the ability to perform tasks involving both the wrist and hand. Future studies will include using the system proposed here to control a partial-hand prosthesis in real-time, using quantifiable metrics such as the Box and Block Test (Cromwell, 1976) and the Southampton Hand Assessment Procedure (Light et al., 2002), to evaluate user performance.

## Authors contributions

EE helped in algorithm development; collecting, analyzing, and interpreting the data; and drafting the manuscript. LH helped in conceiving the study concept, algorithm development, interpreting the data, drafting the manuscript, critically revising the manuscript for important intellectual content, obtaining funding, and supervising the study. TK helped in conceiving the study concept, interpreting the data, critically revising the manuscript for important intellectual content, and obtaining funding. All authors read and approved the final manuscript.

### Conflict of interest statement

The authors declare that the research was conducted in the absence of any commercial or financial relationships that could be construed as a potential conflict of interest. LH and TK have ownership interest in Coapt LLC., a start-up company that sells myoelectric pattern recognition control systems. No Coapt products were used as part of this research.

## Abbreviations

EMG: Electromyogram
ADL: Activity of daily living
VR: Virtual reality
LDA: Linear discriminant analysis
ANOVA: Analysis of variance.
